# The dilution effect of healthy lifestyles on the risk of cognitive function attributed to socioeconomic status among Chinese older adults: A national wide prospective cohort study

**DOI:** 10.7189/jogh-14-04010

**Published:** 2024-02-02

**Authors:** Yao Li, Yuhong Tang, Jiaping Lu, Hengjing Wu, Longbing Ren

**Affiliations:** 1 Clinical Centre for Intelligent Rehabilitation Research, Shanghai YangZhi Rehabilitation Hospital, Shanghai Sunshine Rehabilitation Centre, School of Medicine, Tongji University, Shanghai, China; 2 Department of Endocrinology, Qingpu Branch of Zhongshan Hospital, Fudan University, Shanghai, China; 3 China Centre for Health Developments, Peking University, Beijing, China

## Abstract

**Background:**

Lower socioeconomic status (SES) is a risk factor for poor cognitive function, while a healthy lifestyle is associated with better cognitive function. We examined the complex relationship between SES and a healthy lifestyle and cognitive function among older Chinese adults.

**Methods:**

We used a national prospective cohort of the Chinese Longitudinal Healthy Longevity Survey (CLHLS) from 2008–18, aged 65 years and older with normal cognition at baseline. Participants were categorised into the favourable group if they had four to six healthy lifestyle factors and the unfavourable group for zero to three factors. SES was classified as higher and lower by assessing the socioeconomic vulnerability index (SEVI) with six components. Cognitive function was measured using the Mini-Mental State Examination (MMSE) scores and the standardised Z-scores. We applied the linear mixed effects and time-dependent Cox regression models to explore associations and further stratified the analysis by healthy lifestyles.

**Results:**

A total of 6851 participants were included (the mean age was 80.87, 43.44% had a favourable lifestyle, and 49.29% had higher SES). Over the 10-year follow-up period, SES status and lifestyle profiles significantly affected the decline in the standardised Z-scores (*P <* 0.05). The higher SES group with favourable lifestyles exhibited a slower cognitive decline than those with lower SES (by 0.031 points per year, *P* < 0.05). The association was not observed in those in the unfavourable group (0.010 points per year, *P* > 0.05). During a follow-up, 25.06% of participants developed cognitive impairment (MMSE<18). We also observed a significant interaction between SES and healthy lifestyles (*P* < 0.05), with the corresponding associations of SES being more pronounced among participants with unfavourable lifestyles (hazard ratio (HR) = 0.821; 95% confidence interval (CI) = 0.701–0.960) than those with favourable lifestyles (HR = 1.006; 95% CI = 0.844–1.200).

**Conclusions:**

A healthy lifestyle may attenuate the adverse impacts of lower SES on cognitive function among older adults. This study might provide important information for protecting cognitive function, especially in low- and middle-income countries.

With greater life expectancy, many more older people will be predisposed to the risk of cognitive impairment, characterised by a decline in memory, attention, language, and other cognitive functions as a transitional state between normal ageing and dementia, which imposed the massive burden of diseases on individuals and their caregivers [1,2]. Therefore, identifying modifiable risk factors for cognitive decline and its most vulnerable groups is crucial for developing prevention strategies, given the lack of effective treatment for cognitive impairment.

World Health Organization (WHO) estimations show that by 2050, 80% of older adults worldwide will reside in low- and middle-income countries [3], but only a few epidemiological studies on cognitive function in low- and middle-income countries have been conducted in nationally representative samples of older adults. Several studies elaborated on the relationship between socioeconomic status (SES) and cognitive function, centring on education level [4–6]. Previous studies used single variables (e.g. income and education level) to represent individual-level SES [4–6]. It is essential to construct a comprehensive SES variable comprising different aspects of SES [7]. Furthermore, preventing cognitive impairment through lifestyle modifications has gained considerable attention in recent years, as there is growing evidence that they help slow cognitive decline and may reduce the risk of cognitive impairment [8,9]. However, many of these factors (e.g. diet and exercise) are likely to have synergistic effects on cognition risk, the overall effect of multiple modifiable lifestyle factors should be examined in combination [8–11]. Despite some findings implicating that a healthy lifestyle might alleviate the socioeconomic inequities in health due to an interactive effect [12,13], how these factors interact, and their overall effect on cognitive function are poorly understood. As a developing country with the largest older population, much of China’s population lives in low- and middle-income countries; the differences in income, education, and health care accessibility contribute to significant health inequalities, making it necessary to assess the impact of multiple lifestyles and SES on cognitive functioning in the older population, which could provide some assistance to policymakers in developing health policies to improve the cognitive health of the entire older population [14,15].

Given the positive association between a combined healthy lifestyle and cognitive function and the opposite association between lower SES and cognitive function, it is plausible to hypothesise that it may modify the association between SES and cognitive function. Thus, we aimed to explore their interaction with cognitive function (cognitive decline and cognitive impairment) among older adults.

## METHODS

### Study population

The China Longitudinal Healthy Longevity Survey (CLHLS) is a nationwide, population-based cohort study that recruits older participants from 22 provinces using a multistage whole-group sampling method. A more detailed description of the sampling design can be found elsewhere [16]. The survey was initiated in 1998, with follow-up conducted every two to three years. Written informed consent from participants was obtained at baseline, and each follow-up and the study was approved by the Biomedical Ethics Committee of Peking University, China (IRB00001052-13074). Our study enrolment procedure began in the 2008 wave and comprised four sequential waves (lasting about 10 years together). A total of 6851 cognitively normal participants were enrolled at baseline after eligibility screening based on the exclusion criteria: under 65 years old, with poor cognitive function (Mini-Mental State Examination (MMSE)<18) at baseline, with missing values in the MMSE, die or lost follow-up in the second survey (Figure S1 in the **Online Supplementary Document**).

### Assessment of cognitive function

We utilised the Chinese version of the MMSE to assess participants’ cognitive function, which has been verified to have good reliability and validity [17,18]. It assesses cognitive function in five dimensions with 24 items, including orientation, registration, attention and calculation, recall, and language (Table S1 in the **Online Supplementary Document**). Consistent with previous studies [19,20], participants who scored less than 18 in the MMSE were defined to have cognitive impairment. Further, we conducted a secondary analysis using MMSE scores as a continuous variable.

We used a standardised Z-score of MMSE scores at each wave to evaluate the rate of cognitive decline. First, we calculated the Z-score by subtracting the participant’s score from the average and dividing it by the standard deviation (SD). We calculated a composite global cognitive Z-score for each participant by averaging the Z-scores of the five dimensions and re-standardised this using a composite global cognitive Z-score at baseline to generate a standardised Z-score. A standardised Z-score of one at any given wave indicated that the score was one SD higher than the mean global cognitive Z-score at baseline [21].

### Assessment of healthy lifestyle

We collected lifestyle information at baseline and each follow-up through a healthy behaviour questionnaire. Healthy lifestyle status was assessed by six lifestyle factors: drinking, smoking, physical exercise, diet, cognitive activity, and social contact [11]. For smoking, participants were categorised as current smokers, never smoking, or used to smoke, and never smoking was deemed a healthy lifestyle factor. Using a similar evaluation, we defined never, former, and current drinkers and physical exercisers. For diet, the frequency of 13 food items consumed by participants was recorded (fruits, vegetables, fish, meat, dairy products, vegetable oil, eggs, whole grain, legumes, nuts, tea, garlic and mushrooms, and algae), and at least seven appropriate weekly amounts were considered healthy. Participation at least once weekly was considered healthy for cognitive activity (writing, reading, playing cards, mahjong, watching TV and listening to the radio) and social contact (organised activities). We defined a favourable lifestyle as approximately the top 40% of healthy factors in the cohort distribution [11,22]. Ultimately, we divided all participants into favourable (four to six healthy factors) and unfavourable (zero to three) groups based on the combined effect of lifestyle factors.

### Assessment of socioeconomic status

Socioeconomic status (SES) was assessed by the socioeconomic vulnerability index (SEVI) with six components [23], collected at baseline and at each follow-up. For educational attainment, zero years of schooling scored 1, one to six years  scored 0.5, and seven or more years of schooling scored 0. White collar (professional technician, doctor, teacher, office worker, and military) scored 0 for primary occupation and other types 1. For economic independence, daily expenses paid primarily by one’s salary or pension scored 0 (otherwise 1). For family economic status, a score of 0 for very rich, 0.25 for rich, 0.5 for general, 0.75 for poor, and 1 for very poor. For health care services, a score of 0 for timely access and 1 for otherwise. For urban-rural residences, a score of 0 was assigned to urban, 0.5 to urban, and 1 to rural. We obtained the SEVI score by dividing the total score of the above variables by six, ranging from zero to one, with higher scores indicating lower levels of SES. For further analysis, participants were categorised into lower (≤0.667) and higher (>0.667) SES groups based on the median of the SEVI scores.

### Covariates

According to relevant references [11,19,24], sociodemographic information and health-related indicators were considered to control the potential bias. The covariates include age, gender (male or female), region (east, centre, or west), and marital status (married, widowed, divorced/separated/single). We collected health-related covariates at baseline and each follow-up, including body mass index (BMI), basic activity of daily living (BADL), instrumental activity of daily living (IADL), and medical illnesses. Medical illnesses were based on self-reported hypertension, diabetes, heart disease, stroke and other cerebrovascular, and dyslipidaemia.

### Statistical analysis

Assuming a ratio of 40:60 for the size of people in the favourable vs unfavourable lifestyle group, with a *P*-value <0.05, a power of 90%, an odds ratio (OR) = 0.7, and a cognitive impairment incidence of 31% in the favourable group [11,19], we calculated the sample size to be at least 598 for the favourable group and 895 for the unfavourable group. Continuous variables were presented as means and SD if distributed normally by the Kolmogorov-Smirnov test. Otherwise, medians and interquartile ranges were applied. Numbers and proportions presented categorical variables. Baseline characteristics were compared between the unfavourable and favourable groups using an independent sample *t* test for continuous variables if distributed normally, the Mann-Whitney U test if not, and χ^2^ tests or Fisher exact tests for categorical variables. The numbers of missing values are summarised in Table S2 in the **Online Supplementary Document**. We used a multiple imputation chain equation to impute missing data, analysed five imputed data sets separately, and then combined the results using Rubin’s method.

We used linear mixed-effects models to assess the longitudinal association between a healthy lifestyle and SES, as well as their interaction and cognitive decline, and all statistical assumptions were tested before the interpretation of results. A standardised Z-score of MMSE scores was the dependent variable; the fixed effects included lifestyle profiles, SES, follow-up year from baseline (time), interaction of lifestyle and time (healthy lifestyle group × time), and interaction of SES and time (SES group × time), and the random effects included intercept and time. MMSE score at baseline and other covariates (age, gender, region, marital status, BMI, BADL disability, IADL disability, and medical illnesses) were also adjusted in this model. Further, we added ‘healthy lifestyle × SES’ to the fixed effects to explore interactions, including all the variables in step one. If there was an interaction, we stratified the analysis by two healthy lifestyle groups, with all variables in the first step only excluding the ‘healthy lifestyle group × time’ in fixed effects. In addition, the same analysis was performed using MMSE scores as the dependent variable. Based on previous research [11], we performed time-dependent Cox regression models to calculate hazard ratios (HRs) and 95% confidence intervals (95% CIs) for lifestyle and SES on the progression of cognitive impairment in the total population and healthy lifestyle stratified population.

We conducted additional sensitivity analyses to evaluate the robustness of our findings. We included the number of lifestyle factors as a continuous variable in the mixed and Cox models. Further, we evaluated the association of each single lifestyle factor and SES factor with cognitive function in two models. We plotted three knots cubic splines to explore the nonlinearity of the number of lifestyle factors and SEVI with risks of developing cognitive impairment to test the rationale of our grouping method. Main analyses were repeated and stratified by age groups (<80 and ≥80), gender (male and female), and marital status (married and unmarried, widowed, divorced, separated, or single) to test the robustness and potential variations in different subgroups. Considering the relatively high number of deaths and drop-out visits due to the high age of our participants, we used an inverse probability weighting model to assess whether withdrawal from the study affected the effects of lifestyle on cognitive impairment, and a competing risk model was built for evaluated the bias caused by competing risk from death.

All statistical analysis was conducted by using R, version 4.2.3 (R Core Team, Vienna, Austria). We used the following packages: ‘mice’ for imputing, ‘lmerTest’ for linear mixed effects models, ‘survival’ for time-dependent Cox regression models, ‘cmprsk’ for competing risk models, and ‘ipw’ for inverse probability weighting. Statistical significance was defined by *P* < 0.05 in two-sided testing.

## RESULTS

### Study participants’ characteristics

A total of 6851 CLHLS participants with normal cognitive function at baseline were enrolled in our study. Nearly half (51.15%) of participants were female, with age x̄ = 80.87 (SD = 10.10), 43.44% had a favourable lifestyle, and 49.29% were in the higher SES group. Compared to the unfavourable group, the participants were more likely to be younger, female, married, have higher BMI, lower SEVI, and have more chronic disease (**Table 1**).

**Table 1:** Baseline characteristics of two healthy lifestyle groups*

Characteristics	Total	Unfavourable group	Favourable group	*P*-value
Age in years, x̄ (SD)	80.87 (10.10)	81.54 (10.13)	80.00 (10.00)	<0.05
Gender				<0.05
*Male*	3347 (48.85)	1990 (51.35)	1357 (45.60)	
*Female*	3504 (51.15)	1885 (48.65)	1619 (54.40)	
Region				<0.05
*East*	3229 (47.13)	1617 (41.73)	1612 (54.17)	
*Centre*	1729 (25.24)	1052 (27.15)	677 (22.75)	
*West*	1893 (27.63)	1206 (31.12)	687 (23.08)	
Marital status				<0.05
*Married*	3133 (45.73)	1660 (42.84)	1473 (49.50)	
*Widowed, divorced, separated, or single*	3718 (54.27)	2215 (57.17)	1503 (50.51)	
BMI in kg/m2, x̄ (SD)	21.24 (12.15)	20.18 (8.94)	21.61 (13.06)	<0.05
MMSE score, x̄ (SD)	26.78 (3.27)	26.35 (3.43)	27.34 (2.95)	<0.05
BADL disability	394 (5.75)	203 (5.24)	191 (6.42)	<0.05
IADL disability	3275 (47.80)	1984 (51.20)	1291 (43.38)	<0.05
Medical illnesses				
*Hypertension*	1518 (22.16)	739 (19.07)	779 (26.18)	<0.05
*Diabetes*	207 (3.02)	60 (1.55)	147 (4.94)	<0.05
*Heart disease*	654 (9.55)	271 (6.99)	383 (12.87)	<0.05
*Stroke, cerebrovascular disease*	358 (5.23)	155 (4.00)	203 (6.82)	<0.05
*Dyslipidaemia*	121 (1.77)	46 (1.19)	75 (2.52)	<0.05
Healthy lifestyle factors				
*Healthy diet*	3646 (53.22)	1156 (29.83)	2490 (83.67)	<0.05
*Never smoking*	5352 (78.12)	2626 (67.77)	2726 (91.60)	<0.05
*Regular physical exercise*	2469 (36.04)	634 (16.36)	1835 (61.66)	<0.05
*Never drinking*	5439 (79.39)	2686 (69.32)	2753 (92.51)	<0.05
*Active cognitive activity*	5186 (75.70)	2302 (59.41)	2884 (96.91)	<0.05
*Active social contact*	404 (5.90)	36 (0.93)	368 (12.37)	<0.05
The number of healthy lifestyle factors, x̄ (SD)	3.28 (1.16)	2.44 (0.70)	4.39 (0.57)	<0.05
Socioeconomic status group				<0.05
*Lower*	3474 (50.71)	2335 (60.26)	1139 (38.27)	
*Higher*	3377 (49.29)	1540 (39.74)	1837 (61.73)	
Years of education				<0.05
*<1*	3587 (52.36)	2243 (57.88)	1344 (45.16)	
*1–6*	2388 (34.86)	1290 (33.29)	1098 (36.90)	
*>6*	876 (12.79)	342 (8.83)	534 (17.94)	
Occupation				<0.05
*White collar*	671 (9.79)	209 (5.39)	462 (15.52)	
*Other types*	6180 (90.21)	3666 (94.61)	2514 (84.48)	
Economic independence				<0.05
*One’s own*	2391 (34.90)	1090 (28.13)	1301 (43.72)	
*Others*	4460 (65.10)	2785 (71.87)	1675 (56.28)	
Economic status				<0.05
*Very rich*	77 (1.12)	24 (0.62)	53 (1.78)	
*Rich*	888 (12.96)	352 (9.08)	536 (18.01)	
*General*	4761 (69.49)	2693 (69.50)	2068 (69.49)	
*Poor*	951 (13.88)	668 (17.24)	283 (9.51)	
*Very poor*	174 (2.54)	138 (3.56)	36 (1.21)	
Timely access to health care services				<0.05
*Yes*	6432 (93.88)	3540 (91.35)	2892 (97.18)	
*No*	419 (6.12)	335 (8.65)	84 (2.82)	
Place of residence				<0.05
*Urban*	1226 (17.90)	319 (8.23)	907 (30.48)	
*Town*	1446 (21.11)	759 (19.59)	687 (23.08)	
*Rural*	4179 (61.00)	2797 (72.18)	1382 (46.44)	
SEVI, x̄ (SD)	0.59 (0.19)	0.64 (0.16)	0.52 (0.21)	<0.05

BADL – basic activity of daily living, BMI – body mass index, IADL – instrumental activity of daily living, MMSE – Mini-Mental State Examination, SD – standard deviationSEVI – socioeconomic vulnerability index, x̄ – mean

*Presented as n (%) unless specified otherwise.

### Lifestyle and cognitive decline

**Figure 1:**
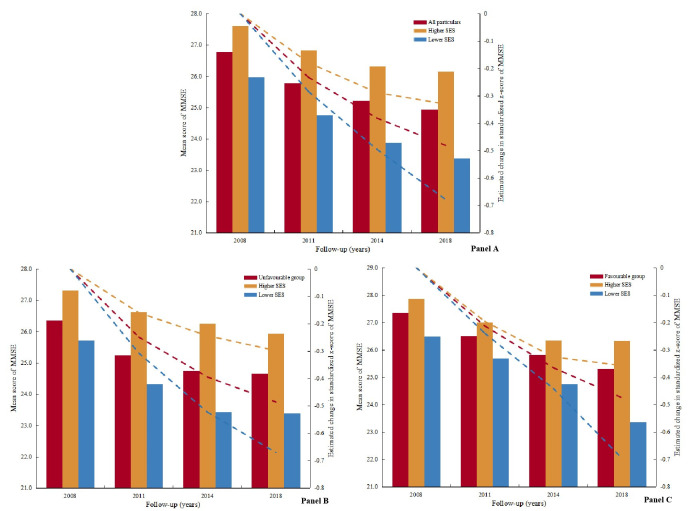
Longitudinal change in the mean score of MMSE and standardised Z-score of MMSE among different groups over 10 years. **Panel A.** Stratified by SES in all particular. **Panel B.** Stratified by SES in the unfavourable lifestyle group. **Panel C.** Stratified by SES in the favourable lifestyle group.

We used LMMs to examine the influence of healthy lifestyle and SES on the annual rate of change in cognitive function over the 10-year follow-up period. Lifestyle and SES were associated with MMSE scores and standardised Z-scores (**Table 2**). Compared with the unfavourable group, the decline in MMSE scores occurred slower in the favourable group (0.187 points per year), and participants had decelerated cognitive decline (a standardised Z-score) of 0.038 SD per year. Furthermore, the rate of decline in MMSE scores was slower in the higher SES than in the lower SES group (0.057 points per year), and participants of the higher SES had a decelerated cognitive decline of 0.013 SD per year (**Figure 1**, panel A). We observed the significant interactions between lifestyle groups and SES for both MMSE scores (*P* < 0.05) and standardised Z-scores (*P* < 0.05) (Table S4 in the **Online Supplementary Document**), with the corresponding associations of SES being much more pronounced among participants with unfavourable lifestyle, compared to those with favourable lifestyle (**Figure 1**, Panels B–C and **Table 2**).

**Table 2:** Associations of healthy lifestyle and socioeconomic status with cognitive decline in a population stratified by healthy lifestyle using Rubin’s method

	Estimate*	SE	*T-*value	*P-*value
**All participants MMSE score**				
Healthy lifestyle group × time				
*Unfavourable × time*	ref			
*Favourable × time*	0.187	0.018	10.117	<0.05
Socioeconomic status group × time				
*Lower × time*	ref			
*Higher × time*	0.057	0.020	2.851	<0.05
**All participants standardised Z-score**				
Healthy lifestyle group × time				
*Unfavourable × time*	ref			
*Favourable × time*	0.038	0.003	10.914	<0.05
Socioeconomic status group × time				
*Lower × time*	ref			
*Higher × time*	0.013	0.004	3.348	<0.05
**Unfavourable group MMSE score**				
Socioeconomic status group × time				
*Lower × time*	ref			
*Higher × time*	0.090	0.027	3.340	<0.05
**Unfavourable group standardised Z-score**				
Socioeconomic status group × time				
*Lower × time*	ref			
*Higher × time*	0.031	0.006	5.293	<0.05
**Favourable group MMSE score**				
Socioeconomic status group × time				
*Lower × time*	ref			
*Higher × time*	0.035	0.030	1.171	>0.05
**Favourable group standardised Z-score**				
Socioeconomic status group × time				
*Lower × time*	ref			
*Higher × time*	0.010	0.006	1.842	>0.05

MMSE – Mini-Mental State Examination, ref. – reference, SE – standard error

*Adjusted covariates of linear mixed effect model included MMSE score at baseline, age, gender, region, marital status, BMI, BADL disability, IADL disability, hypertension, diabetes, heart disease, stroke and cerebrovascular disease, and dyslipidaemia.

The results of the main analysis were consistent with the results of the sensitivity analysis by using the lifestyle factors and SEVI as continuous variables (Tables S4–6 in the **Online Supplementary Document**). We evaluated the contribution of each lifestyle, and the SES component was also evaluated. The results showed that a healthy diet, regular physical activity, active cognitive activity, years of education, economic independence, economic status (very poor), timely access to health care services, and place of residence were associated with the annual rate of change in MMSE scores and standardised Z-scores (Table S7–9 in the **Online Supplementary Document**). The subgroup results of two age groups, male and married, were similar to the main analyses. However, SES was not associated with cognitive decline in the female and unmarried groups.

### Lifestyle and risk of cognitive impairment

**Figure 2:**
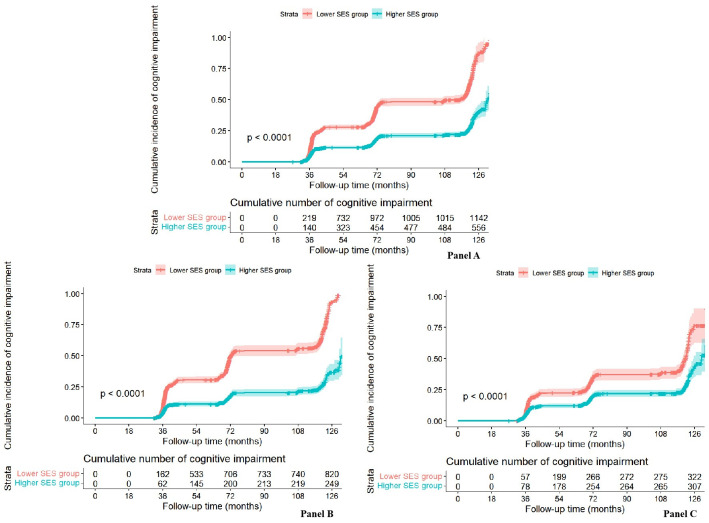
Kaplan-Meier curves for the overall cumulative incidence of cognitive impairment and number at risk. The shaded area indicates the range of 95% CIs for the corresponding cumulative incidence curve. *P*-value indicates the significance level from comparing incidence curves using the Log-rank test. **Panel A.** Stratified by SES in all particulars. **Panel B.** Stratified by SES in the unfavourable lifestyle group. **Panel C.** Stratified by SES in the favourable lifestyle group.

After an average of 76.15 months of follow-up, 1717 of 6851 participants (25.06%) suffered possible cognitive impairment (Table S10 in the **Online Supplementary Document**). For the Kaplan-Meier curves for the overall cumulative incidence and number at risk for cognitive impairment stratified by the SES group, the analysis showed significant differences in the above cumulative incidence curves (*P* < 0.05) (**Figure 2**). The time-dependent Cox regression model suggested that favourable lifestyles and high SES were associated with a lower probability of progression to cognitive impairment among all participants over 10 years of follow-ups (**Table 3**). There was also a significant interaction between the lifestyle group and SES for cognitive decline (*P* < 0.05) (Table S11 in the **Online Supplementary Document**). Similarly, the corresponding association for SES was more pronounced in participants with unfavourable lifestyles than those with favourable lifestyles (**Table 3**).

**Table 3:** HRs of healthy lifestyle and socioeconomic status on the onset of cognitive impairment in a population stratified by healthy lifestyle using Rubin’s method

	HR (95% CI)*	*P*-value
**All participants**		
Healthy lifestyle group		
*Unfavourable*	ref	
*Favourable*	0.905 (0.833–0.983)	<0.05
Socioeconomic status group		
Lower	ref	
Higher	0.762 (0.697–0.832)	<0.05
**Unfavourable group**		
Socioeconomic status group		
*Lower*	ref	
*Higher*	0.821 (0.701–0.960)	<0.05
**Favourable group**		
Socioeconomic status group		
*Lower*	ref	
*Higher*	1.006 (0.844–1.200)	>0.05

CI – confidence interval, HR – hazard ratio, ref –reference

*Adjusted covariates of linear mixed effect model included MMSE score at baseline, age, gender, region, marital status, BMI, BADL disability, IADL disability, hypertension, diabetes, heart disease, stroke and cerebrovascular disease, and dyslipidaemia.

The result was consistent when considering the lifestyle factors and SEVI as a continuous variable. We evaluated the relationship between each lifestyle and SES factor; the results displayed that regular physical activity, never drinking, active cognitive activity, active social contact, years of education, economic independence, and place of residence were associated with cognitive impairment. A significant nonlinear relationship was observed for both lifestyle factors and SEVI (*P* < 0.05). The results of subgroups displayed that the associations of SES and cognitive impairment for female and unmarried groups differed substantially from those of the main analyses. The results were also similar when considering the competing risk of death or participants who withdrew from the study (Figure S2–3 and Tables S12–14 in the **Online Supplementary Document**).

## DISCUSSION

In this study, we presented the complex effects of lifestyle and SES on cognitive function in a sample of Chinese adults aged 65. We found older adults with higher SES had slower cognitive decline and a 23.8% lower risk of developing cognitive impairment. However, the associations were significantly modified by the healthy lifestyle. The higher SES group with favourable lifestyles exhibited the corresponding associations than those with lower SES, but the associations were not observed in those in the unfavourable group.

Inequality in SES is one of the important risk factors for early morbidity and mortality. Studies from high-income countries have shown that the risk of cardiovascular disease is 2.5 times higher, and the risk of death is 2.13 times higher in low-SES than in high-SES populations [25]. Furthermore, older adults with lower SES in low- and middle-income countries have a higher risk [11] of disease and death due to excessive pollution, low levels of medical care, poor drug accessibility, unhealthy lifestyles, and ageing trends [26,27]. Previous studies have shown the harm of lower SES on cognitive health in the older Chinese population [5,28], and our study validated the risk of SES on cognitive function and improved the reliability of the evidence by including more SES indicators. The above findings suggest an urgent need to find ways to reduce the health inequalities associated with SES.

Lifestyle factors are potentially modifiable, and lifestyle-based interventions can reduce the risk of cognitive impairment in older age. Several previous studies have shown that lifestyles such as smoking, physical activity, healthy diet, and cognitive activity are associated with the rate of cognitive decline in later life [29]. However, synergistic effects exist among multiple lifestyles, and evidence for effects on cognitive function based on multiple lifestyle assessments remains limited. Recent evidence suggests that a greater variety of healthy lifestyles is associated with slower memory decline in an older Chinese population [11], consistent with our findings. Furthermore, a systematic review of 65 randomised controlled trials showed that exercise was the most promising lifestyle intervention for improving various cognitive functions in people with mild cognitive impairment and dementia. Still, the effectiveness of diet interventions was not examined due to the lack of randomised controlled trials on a healthy dietary pattern [30]. Another systematic review that included 27 observational cohort studies found that eating a healthy diet and participating in leisure and physical activities may prevent cognitive decline and cognitive impairment in older adults regardless of apolipoprotein E genotype, and a combination of lifestyles may have a multiplier effect compared with individual factors [31]. At the same time, there is a complex relationship between lifestyle and SES, and recent studies have suggested that SES may influence lifestyle, which can lead to differences in the occurrence of adverse cognitive outcomes. A meta-analysis including 31 studies indicated that lifestyle explained over 20% of the risk of health outcomes attributable by SES, suggesting that adherence to a healthy lifestyle may partially offset the harms of socioeconomic inequalities [7]. However, studies based on the National Health and Nutrition Examination Survey showed no interaction between lifestyle and SES [24], which may be related to the type of lifestyle assessed and the economic level of the region. In our study, we showed a significant interaction between a comprehensive lifestyle assessment based on smoking, alcohol consumption, diet, exercise, cognitive activity, and social activity and SES on cognitive function in the older Chinese population, and subgroup analyses suggested that a healthier lifestyle could offset some of the cognitive risk associated with SES. This is consistent with the results of some studies [27,32] and emphasises the need for lifestyle changes in the older population. In addition, subgroup analysis showed that female and unmarried participants received weaker effects of socioeconomic inequality. One possible reason is that females and unmarried participants were more concentrated at low SES levels (both groups having as much as 65% lower socioeconomic rates). However, the exact reason for this difference still needs further investigation.

The main strength of this study is that the results were obtained from a nationwide representative longitudinal cohort of older people in China. The large sample size can allow for joint and stratified analyses with sufficient statistical power and provide an opportunity to observe the longitudinal change of cognitive function in older adults. We established a lifestyle and SES evaluation system with more indicators to evaluate their complex relationship with cognitive function comprehensively. In addition, a series of sensitivity analyses were conducted to show the robustness of the results.

Our study also had several limitations. The lifestyle and SES factors assessments were based on self-reports and are prone to measurement errors despite strict collection standards. Further, death, lost visits, and missing data could lead to the exclusion of some subjects, which could lead to selection bias, but the corresponding sensitivity analysis showed consistent results. Given the nature of our study design, we could not evaluate whether lifestyle had already begun to affect cognition at the time of enrolment. We used MMSE scores instead of clinical diagnosis to determine cognitive function, but the adapted Chinese version was demonstrated to be reliable and valid in prior research. Further, as in most large population cohort studies, we used the time until the follow-up event rather than the true time to event, which could overestimate the relationship between lifestyle and cognitive function. Also, all residual confounding factors cannot be eliminated due to limited covariates, such as lack of genotype information or other diseases. However, the results have adjusted for individual characteristics and major comorbidities.

## CONCLUSIONS

Our findings indicated that adhering to favourable lifestyles might benefit cognitive function induced by long-term lower SES exposure in older adults. These results might offer important information for public health initiatives to protect older adults against cognitive decline, especially in low- and middle-income countries.

## Additional material


Online Supplementary Document.
