# Supplementary material for: The dilution effect of healthy lifestyles on the risk of cognitive function attributed to socioeconomic status among Chinese older adults: A national wide prospective cohort study
Source: J Glob Health. 2024 Feb 2;14:04010. doi: 10.7189/jogh-14-04010 (PMC10835516; doi:10.7189/jogh-14-04010)
Supplement: Online Supplementary Document. [file jogh-14-04010-s001.pdf]

Supplementary appendix

This appendix has been provided by the authors to give readers additional information about their working figures and tables.

CONTENT

Supplementary table 1. The Chinese version of the mini-mental state examination adopted in the CLHLS. 1
Supplementary table 2. Distribution of missing values with different follow-up years 2
Supplementary table 3. Baseline characteristics of two socioeconomic status groups. 3
Supplementary table 4. Interaction between healthy lifestyle and socioeconomic status on cognitive decline using Rubin's method. 5
Supplementary table 5. Associations of the number of healthy lifestyle factors and SEVI with cognitive decline using Rubin's method. 7
Supplementary table 6. Association between SEVI and cognitive decline using Rubin's method, stratified by healthy lifestyle. 8
Supplementary table 7. Association of each lifestyle and SES factor and cognitive decline using Rubin's method. 9
Supplementary table 8. Associations of healthy lifestyle and socioeconomic status with cognitive decline using Rubin's method: subgroup analyses. 12
Supplementary table 9. Association between socioeconomic status and cognitive decline using Rubin's method, stratified by healthy lifestyle: subgroup analyses. 14
Supplementary table 10. Baseline characteristics of participants by follow-up cognitive impairment. . 16
Supplementary table 11. Interaction between healthy lifestyle and socioeconomic status on the onset of cognitive impairment using Rubin's method. 18
Supplementary table 12. Hazard ratios of SEVI on the onset of cognitive impairment in a population stratified by healthy lifestyle using Rubin's method. 19
Supplementary table 13 Hazard ratios of socioeconomic status on the onset of cognitive impairment in a population stratified by healthy lifestyle using Rubin's method: subgroup analyses. 20
Supplementary table 14. Association of healthy lifestyle and socioeconomic status with cognitive impairment by adjusting with the death as the competing risk and using the inverse probability weighting method. 21
Supplementary figure 1. Flow chart of the included CLHLS individuals. 22
Supplementary figure 2. Hazard ratios of each lifestyle and socioeconomic status factor on the onset of cognitive impairment 23
Supplementary figure 3. Cubic splines of the number of healthy lifestyle factors and SEVI on the risk of cognitive impairment. 24

Supplementary table 1. The Chinese version of the mini-mental state examination adopted in the CLHLS.

Domain	Question	Score (Total = 30)
Orientation	What time of day is it right now (morning, afternoon, evening)?	1
	What is the animal year of this year?	1
	What is the date (day and month) of the mid-autumn festival?	1
	What is the season right now?	1
	What is the name of this county or district?	1
	Please name as many kinds of food as possible in 1 minute (1 point for each food and 7 points for those who name 7 or more foods).	7
Registration	Table (1 point), apple (1 point), cloth (1 point). Please repeat these 3 objects.	3
Attention and calculation	I will ask you to spend $3 from $20, then you must spend $3 from the number you arrived at and continue to spend $3 until you are asked to stop (1 point for each calculation).	5
	The individual is asked to draw a figure of overlapping pentagons.	1
Recall	Name the 3 objects learned earlier (table, apple, and cloth).	3
Language	Naming pen (1 point) and watch (1 point).	2
	Repeating the following sentence: “What you plant is what you will get.”	1
	The individual is asked to follow the interviewer’s instruction: “Take the paper using your right hand (1 point), fold it in the middle using both hands (1 point), and place the paper on the floor (1 point).”	3

Note: We scored each question as zero (wrong or unable to answer) or one (correct) , and the scores ranged between 0 and 30, with a higher score implying better cognitive performance.

Supplementary table 2. Distribution of missing values with different follow-up years

Characteristics	2008(n=6851)	2011(n=6851)	2014(n=4037)	2018(n=1989)
Region	0.39%	0.72%	0.40%	0.70%
Marital status	0.00%	0.31%	0.89%	0.90%
BMI (kg/m2)	0.00%	2.72%	2.77%	2.08%
BADL	0.00%	2.17%	3.15%	2.92%
IADL	0.00%	0.31%	0.87%	1.01%
Hypertension	0.01%	1.10%	2.24%	2.34%
Diabetes	0.01%	1.36%	2.08%	2.76%
Heart disease	0.01%	1.11%	1.56%	1.92%
Stroke, cerebrovascular disease	0.01%	1.79%	1.88%	1.51%
Dyslipidemia	0.03%	1.48%	1.92%	2.78%
Healthy diet	0.10%	0.72%	2.55%	2.58%
Never smoking	0.00%	0.34%	0.45%	1.11%
Regular physical exercise	0.00%	0.79%	2.58%	2.21%
Never drinking	0.00%	0.95%	0.64%	2.01%
Active cognitive activity	0.00%	0.13%	0.30%	0.50%
Active social contact	0.00%	0.22%	0.54%	1.51%
Years of education	0.23%	0.23%	0.40%	0.80%
Occupation	0.04%	0.04%	0.07%	0.15%
Economic independence	0.00%	0.23%	0.89%	1.57%
Economic status	0.00%	0.80%	0.79%	1.11%
Timely access to healthcare services	0.00%	0.53%	0.72%	1.61%

Supplementary table 3. Baseline characteristics of two socioeconomic status groups.

**Characteristics**	**Lower group (n=3474)**	**Higher group (n=3377)**	**P value**
Age (years)	83.18±9.70	78.50±9.95	<0.05
Gender			<0.05
Male	1232 (35.46%)	2115 (62.63%)	
Female	2242 (64.54%)	1262 (37.37%)	
Region			<0.05
East	1518 (43.70%)	1711 (50.67%)	
Centre	927 (26.68%)	802 (23.75%)	
West	1029 (29.62%)	864 (25.58%)	
Marital status			<0.05
Married	1193 (34.34%)	1940 (57.45%)	
Widowed, divorced, separated, or single	2281 (65.65%)	1437 (42.56%)	
BMI (kg/m^2)	20.57±11.57	21.93±12.69	<0.05
MMSE score	25.96±3.44	27.61±2.85	<0.05
BADL disability	194 (5.58%)	200 (5.92%)	>0.05
IADL disability	2070 (59.59%)	1205 (35.68%)	<0.05
Medical illnesses			
Hypertension	730 (21.01%)	788 (23.33%)	0.025
Diabetes	60 (1.73%)	147 (4.35%)	<0.05
Heart disease	232 (6.68%)	422 (12.50%)	<0.05
Stroke, cerebrovascular disease	140 (4.03%)	218 (6.46%)	<0.05
Dyslipidemia	35 (1.01%)	86 (2.55%)	<0.05
Healthy lifestyle group			<0.05
Unfavourable	2335 (67.21%)	1540 (45.60%)	
Favourable	1139 (32.79%)	1837 (54.40%)	
Healthy lifestyle factors			
Healthy diet	1438 (41.39%)	2208 (65.38%)	<0.05
Never smoking	2895 (83.33%)	2457 (72.76%)	<0.05
Regular physical exercise	856 (24.64%)	1613 (47.76%)	<0.05
Never drinking	2882 (82.96%)	2557 (75.72%)	<0.05
Active cognitive activity	2245 (64.62%)	2941 (87.09%)	<0.05
Active social contact	111 (3.20%)	293 (8.68%)	<0.05
The number of healthy lifestyle factors	3.00±1.06	3.57±1.19	<0.05
Socioeconomic status factors			
Years of education			<0.05
<1	2618 (75.36%)	969 (28.69%)	
1~6	841 (24.21%)	1547 (45.81%)	
＞6	15 (0.43%)	861 (25.50%)	
Occupation			<0.05

White collar	5 (0.14%)	666 (19.72%)	
Other types	3469 (99.86%)	2711 (80.28%)	
Economic independence			<0.05
One's own	96 (2.76%)	2295 (67.96%)	
Others	3378 (97.24%)	1082 (32.04%)	
Economic status			<0.05
Very rich	24 (0.69%)	53 (1.57%)	
Rich	201 (5.79%)	687 (20.34%)	
General	2466 (70.98%)	2295 (67.96%)	
Poor	634 (18.25%)	317 (9.39%)	
Very poor	149 (4.29%)	25 (0.74%)	
Timely access to healthcare services			<0.05
Yes	3084 (88.77%)	3348 (99.14%)	
No	390 (11.23%)	29 (0.86%)	
Place of residence			<0.05
Urban	29 (0.83%)	1197 (35.45%)	
Town	572 (16.47%)	874 (25.88%)	
Rural	2873 (82.70%)	1306 (38.67%)	
SEVI	0.74±0.07	0.44±0.16	<0.05

Supplementary table 4. Interaction between healthy lifestyle and socioeconomic status on cognitive decline using Rubin's method.

	Estimate[a]	Standard Error	T value	P value
**MMSE score**				
Healthy lifestyle group				
Unfavourable	Reference			
Favourable	0.225	0.074	3.027	<0.05
Socioeconomic status group				
Lower	Reference			
Higher	0.139	0.073	1.908	>0.05
Time	-0.928	0.028	-32.852	<0.05
Healthy lifestyle *Socioeconomic status				
Favourable*higher	-0.225	0.090	-2.493	<0.05
Healthy lifestyle group*Time				
Unfavourable*Time	Reference			
Favourable*Time	0.188	0.018	10.149	<0.05
Socioeconomic status group*Time				
Lower*Time	Reference			
Higher*Time	0.056	0.020	2.781	<0.05
[b]Test for linear trend				
Healthy lifestyle factors	-0.157	0.066	-2.394	<0.05
SEVI	-1.300	0.429	-3.028	<0.05
Time	-0.922	0.062	-14.803	<0.05
Healthy lifestyle *SEVI	0.358	0.104	3.445	<0.05
Healthy lifestyle *Time	0.095	0.009	10.711	<0.05
SEVI*Time	-0.362	0.073	-4.976	<0.05
**Standardized Z-score**				
Healthy lifestyle group				
Unfavourable	Reference			
Favourable	0.019	0.014	1.362	>0.05
Socioeconomic status group				
Lower	Reference			
Higher	0.005	0.014	0.356	>0.05
Time	-0.180	0.005	-33.025	<0.05
Healthy lifestyle *Socioeconomic status				
Favourable*higher	-0.045	0.017	-2.641	<0.05
Healthy lifestyle group*Time				
Unfavourable*Time	Reference			
Favourable*Time	0.038	0.003	10.945	<0.05

Socioeconomic status group*Time				
Lower*Time	Reference			
Higher*Time	0.012	0.004	3.275	<0.05
[b]Test for linear trend				
Healthy lifestyle factors	-0.034	0.012	-2.800	<0.05
SEVI	-0.119	0.080	-1.493	>0.05
Time	-0.178	0.012	-14.933	<0.05
Healthy lifestyle factors *SEVI	0.056	0.019	2.888	<0.05
Healthy lifestyle factors *Time	0.019	0.002	11.200	<0.05
SEVI*Time	-0.071	0.014	-5.092	<0.05

[a]Adjusted covariates of linear mixed effect model included MMSE score at baseline, age, gender, region, marital status, BMI, BADL disability, IADL disability, hypertension, diabetes, heart disease, stroke& cerebrovascular disease, and dyslipidemia.

[b]Performed by treating the number of healthy lifestyle factors as a numeric variable.

Supplementary table 5. Associations of the number of healthy lifestyle factors and SEVI with cognitive decline using Rubin's method.

	Estimate[a]	Standard Error	T value	P value
**MMSE score**				
Time	-0.910	0.062	-14.694	<0.05
Healthy lifestyle factors	0.051	0.026	1.948	>0.05
Healthy lifestyle factors*Time	0.094	0.009	10.689	<0.05
SEVI	0.049	0.171	0.284	>0.05
SEVI*Time	-0.380	0.072	-5.252	<0.05
**Standardized Z-score**				
Time	-0.176	0.012	-14.852	<0.05
Healthy lifestyle factors	-0.002	0.005	-0.369	>0.05
Healthy lifestyle factors*Time	0.019	0.002	11.182	<0.05
SEVI	0.091	0.032	2.873	<0.05
SEVI*Time	-0.073	0.014	-5.319	<0.05

[a]Adjusted covariates of linear mixed effect model included MMSE score at baseline, age, gender, region, marital status, BMI, BADL disability, IADL disability, hypertension, diabetes, heart disease, stroke& cerebrovascular disease, and dyslipidemia.

Supplementary table 6. Association between SEVI and cognitive decline using Rubin's method, stratified by healthy lifestyle.

	**Estimate**[a]	**Standard Error**	**T value**	**P value**
**Unfavourable group**				
**MMSE score**				
SEVI	-0.093	0.278	-0.333	>0.05
Time	-0.580	0.076	-7.591	<0.05
SEVI*Time	-0.482	0.109	-4.402	<0.05
**Standardized Z-score**				
SEVI	0.070	0.052	1.353	>0.05
Time	-0.112	0.014	-7.799	<0.05
SEVI*Time	-0.091	0.020	-4.432	<0.05
**Favourable group**				
**MMSE score**				
SEVI	-0.051	0.205	-0.247	>0.05
Time	-0.535	0.060	-8.947	<0.05
SEVI*Time	-0.390	0.093	-4.199	<0.05
**Standardized Z-score**				
SEVI	0.079	0.038	2.094	<0.05
Time	-0.099	0.011	-8.736	<0.05
SEVI*Time	-0.080	0.018	-4.582	<0.05

[a]Adjusted covariates of linear mixed effect model included MMSE score at baseline, age, gender, region, marital status, BMI, BADL disability, IADL disability, hypertension, diabetes, heart disease, stroke& cerebrovascular disease, and dyslipidemia.

Supplementary table 7. Association of each lifestyle and SES factor and cognitive decline using Rubin's method.

	Estimate[a]	Standard Error	T value	P value
**MMSE score**				
Healthy diet (Yes)	0.130	0.059	2.211	<0.05
Never smoking (No)	0.028	0.073	0.380	>0.05
Regular physical exercise (Yes)	-0.047	0.060	-0.782	>0.05
Never drinking (No)	0.118	0.072	1.626	>0.05
Active cognitive activity (Yes)	0.084	0.069	1.207	>0.05
Active social contact (Yes)	0.144	0.116	1.239	>0.05
Years of education				
1~6	0.069	0.097	0.710	>0.05
<1	0.039	0.104	0.378	>0.05
Occupation (Other types)	0.037	0.112	0.331	>0.05
Economic independence (Others)	0.134	0.068	1.957	>0.05
Economic status				
Rich	-0.064	0.256	-0.250	>0.05
General	-0.218	0.248	-0.880	>0.05
Poor	-0.408	0.259	-1.576	>0.05
Very poor	-0.391	0.307	-1.273	>0.05
Timely access to healthcare services (No)	0.022	0.124	0.177	>0.05
Place of residence				>0.05
Town	0.169	0.090	1.875	>0.05
Rural	-0.073	0.084	-0.877	>0.05
Time	-1.100	0.087	-12.610	<0.05
Healthy diet (Yes)*Time	0.065	0.018	3.555	<0.05
Never smoking (No)*Time	-0.060	0.032	-1.883	>0.05
Regular physical exercise (Yes)*Time	0.137	0.019	7.138	<0.05
Never drinking (No)*Time	-0.047	0.027	-1.760	>0.05
Active cognitive activity (Yes)*Time	0.291	0.021	13.852	<0.05
Active social contact (Yes)*Time	0.047	0.035	1.360	>0.05
Years of education*Time				
1~6*Time	-0.069	0.032	-2.110	<0.05
<1*Time	-0.081	0.034	-2.369	<0.05

Occupation (Other types)*Time	-0.004	0.042	-0.101	>0.05
Economic independence (others)*Time	-0.123	0.024	-5.226	<0.05
Economic status*Time				
Rich*Time	0.034	0.063	0.547	>0.05
General*Time	0.037	0.060	0.617	>0.05
Poor *Time	-0.044	0.066	-0.670	>0.05
Very poor*Time	-0.259	0.088	-2.940	<0.05
Timely access to healthcare services (no)*Time	-0.138	0.044	-3.110	<0.05
Place of residence*Time				
Town*Time	0.147	0.034	4.305	<0.05
Rural*Time	0.167	0.034	4.867	<0.05
**Standardized Z-score**				
Healthy diet (Yes)	0.013	0.011	1.146	>0.05
Never smoking (No)	0.003	0.014	0.188	>0.05
Regular physical exercise (Yes)	-0.017	0.011	-1.537	>0.05
Never drinking (No)	0.018	0.014	1.334	>0.05
Active cognitive activity (Yes)	-0.010	0.013	-0.774	>0.05
Active social contact (Yes)	0.019	0.022	0.873	>0.05
Years of education				
1~6	0.025	0.018	1.400	>0.05
<1	0.043	0.019	2.207	<0.05
Occupation (Other types)	0.007	0.021	0.346	>0.05
Economic independence (Others)	0.040	0.013	3.143	<0.05
Economic status				
Rich	-0.007	0.048	-0.140	>0.05
General	-0.029	0.046	-0.625	>0.05
Poor	-0.053	0.048	-1.106	>0.05
Very poor	-0.050	0.057	-0.867	>0.05
Timely access to healthcare services (No)	0.005	0.023	0.199	>0.05
Place of residence				
Town	0.038	0.017	2.240	<0.05
Rural	-0.015	0.016	-0.982	>0.05
Time	-0.206	0.017	-12.478	<0.05
Healthy diet (Yes)*Time	0.014	0.003	4.041	<0.05
Never smoking	-0.011	0.006	-1.783	>0.05

(No)*Time				
Regular physical exercise (Yes)*Time	0.025	0.004	6.974	<0.05
Never drinking (No)*Time	-0.008	0.005	-1.631	>0.05
Active cognitive activity (Yes)*Time	0.056	0.004	14.322	<0.05
Active social contact (Yes)*Time	0.009	0.007	1.392	>0.05
Years of education*Time				
1~6*Time	-0.014	0.006	-2.334	<0.05
<1*Time	-0.018	0.006	-2.805	<0.05
Occupation (Other types)*Time	-0.001	0.008	-0.081	>0.05
Economic independence (others)*Time	-0.022	0.005	-4.986	<0.05
Economic status*Time				
Rich*Time	0.005	0.012	0.392	>0.05
General*Time	0.003	0.011	0.278	>0.05
Poor *Time	-0.013	0.012	-1.048	>0.05
Very poor*Time	-0.053	0.017	-3.213	<0.05
Timely access to healthcare services (no)*Time	-0.025	0.008	-2.979	<0.05
Place of residence*Time				
Town*Time	0.025	0.006	3.876	<0.05
Rural*Time	0.031	0.006	4.774	<0.05

[a]Adjusted covariates of linear mixed effect model included MMSE score at baseline, age, gender, region, marital status, BMI, BADL disability, IADL disability, hypertension, diabetes, heart disease, stroke& cerebrovascular disease, and dyslipidemia.

Supplementary table 8. Associations of healthy lifestyle and socioeconomic status with cognitive decline using Rubin's method: subgroup analyses.

	Age[a]		Gender[b]		Marital status[c]	
	<80 (n=3173)	≥80 (n=3678)	Male (n=3347)	Female (n=3504)	Married (n=3133)	Unmarried (n=3718)
**MMSE score**						
Time	-0.456**	-2.020**	-0.676**	-1.179**	-0.663**	-1.688**
Healthy lifestyle group						
Unfavourable	Reference	Reference	Reference	Reference	Reference	Reference
Favourable	0.207	0.060	0.119	0.061	0.169	0.130
Healthy lifestyle group*Time						
Unfavourable*Time	Reference	Reference	Reference	Reference	Reference	Reference
Favourable*Time	0.127**	0.546**	0.132**	0.250**	0.172**	0.359**
Socioeconomic status group						
Lower	Reference	Reference	Reference	Reference	Reference	Reference
Higher	0.168	0.097	0.077	0.063	0.031	0.139
Socioeconomic status group*Time						
Lower*Time	Reference	Reference	Reference	Reference	Reference	Reference
Higher*Time	0.029*	0.156**	0.068*	0.010	0.082*	0.047
**Standardized Z-score**						
Time	-0.088**	-0.380**	-0.180**	-0.285**	-0.132**	-0.321**
Healthy lifestyle group						
Unfavourable	Reference	Reference	Reference	Reference	Reference	Reference
Favourable	0.013	-0.011	0.006	-0.002	0.008	-0.002
Healthy lifestyle group*Time						
Unfavourable*Time	Reference	Reference	Reference	Reference	Reference	Reference
Favourable*Time	0.026**	0.107**	0.035**	0.067**	0.035**	0.071**
Socioeconomic status group						
Lower	Reference	Reference	Reference	Reference	Reference	Reference
Higher	-0.014	-0.003	-0.015	-0.017	-0.026	0.002
Socioeconomic status group*Time						
Lower*Time	Reference	Reference	Reference	Reference	Reference	Reference
Higher*Time	0.011*	0.032**	0.025**	0.008	0.020**	0.011

[a]Adjusted covariates of linear mixed effect model included MMSE score at baseline, age, gender, region, marital status, BMI, BADL disability, IADL disability, hypertension, diabetes, heart disease, stroke& cerebrovascular disease, and dyslipidemia.

[b]Adjusted covariates of linear mixed effect model included MMSE score at baseline, age, region, marital status, BMI, BADL disability, IADL disability, hypertension, diabetes, heart disease, stroke&

cerebrovascular disease, and dyslipidemia.

[c]Adjusted covariates of linear mixed effect model included MMSE score at baseline, age, gender, region, BMI, BADL disability, IADL disability, hypertension, diabetes, heart disease, stroke& cerebrovascular disease, and dyslipidemia.

Supplementary table 9. Association between socioeconomic status and cognitive decline using Rubin's method, stratified by healthy lifestyle: subgroup analyses.

	Age[a]		Gender[b]		Marital status[c]	
	<80 (n=3173)	≥80 (n=3678)	Male (n=3347)	Female (n=3504)	Married (n=3133)	Unmarried (n=3718)
**Unfavourable group**						
**MMSE score**						
Socioeconomic status						
Lower	Reference	Reference	Reference	Reference	Reference	Reference
Higher	0.095	0.173	0.102	0.054	0.064	0.153
Time	-0.402**	-1.795**	-0.841**	-1.526**	-0.569**	-1.634**
Socioeconomic status*Time						
Lower*Time	Reference	Reference	Reference	Reference	Reference	Reference
Higher*Time	0.069*	0.206**	0.118**	0.051	0.113**	0.062
**Standardized Z-score**						
Socioeconomic status						
Lower	Reference	Reference	Reference	Reference	Reference	Reference
Higher	0.000	0.006	-0.004	-0.006	-0.011	0.006
Time	-0.082**	-0.339**	-0.162**	-0.290**	-0.112**	-0.309**
Socioeconomic status*Time						
Lower*Time	Reference	Reference	Reference	Reference	Reference	Reference
Higher*Time	0.014*	0.043**	0.026**	0.009	0.023**	0.014
**Favourable group**						
**MMSE score**						
Socioeconomic status						
Lower	Reference	Reference	Reference	Reference	Reference	Reference
Higher	0.165	0.057	0.033	0.075	0.003	0.191
Time	-0.340**	-1.834**	-0.929**	-1.169**	-0.620**	-1.476**
Socioeconomic status*Time						
Lower*Time	Reference	Reference	Reference	Reference	Reference	Reference
Higher*Time	0.024	0.036	0.061	0.050	0.085	0.076
**Standardized Z-score**						
Socioeconomic status						
Lower	Reference	Reference	Reference	Reference	Reference	Reference
Higher	-0.016	-0.011	-0.024	-0.015	-0.041	0.011
Time	-0.071**	-0.346**	-0.177**	-0.224**	-0.123	-0.280
Socioeconomic status*Time						
Lower*Time	Reference	Reference	Reference	Reference	Reference	Reference
Higher*Time	0.012	0.021	0.020	0.014	0.023	0.017

[a]Adjusted covariates of linear mixed effect model included MMSE score at baseline, age, gender, region, marital status, BMI, BADL disability, IADL disability, hypertension, diabetes, heart disease, stroke& cerebrovascular disease, and dyslipidemia.

[b]Adjusted covariates of linear mixed effect model included MMSE score at baseline, age, region, marital status, BMI, BADL disability, IADL disability, hypertension, diabetes, heart disease, stroke&

cerebrovascular disease, and dyslipidemia.

[c]Adjusted covariates of linear mixed effect model included MMSE score at baseline, age, gender, region, BMI, BADL disability, IADL disability, hypertension, diabetes, heart disease, stroke& cerebrovascular disease, and dyslipidemia.

Supplementary table 10. Baseline characteristics of participants by follow-up cognitive impairment.

Characteristics	Normal cognition (n=5134)	Cognitive impairment (n=1717)	P value
Age (years)	78.73±9.50	87.28±9.07	<0.05
Gender			<0.05
Male	2768 (53.92%)	579 (33.72%)	
Female	2366 (46.08%)	1138 (66.28%)	
Region			<0.05
East	2508 (48.85%)	721 (41.99%)	
Centre	1273 (24.80%)	456 (26.56%)	
West	1353 (26.35%)	540 (31.45%)	
Marital status			<0.05
Married	2688 (52.36%)	445 (25.92%)	
Widowed, divorced, separated, or single	191 (3.72%)	44 (2.56%)	
BMI (kg/m^2)	21.48±12.07	20.53±12.38	<0.05
MMSE score	27.26±3.00	25.32±3.58	<0.05
BADL disability	197 (3.84%)	197 (11.47%)	<0.05
IADL disability	2045 (39.83%)	1230 (71.64%)	<0.05
Medical illnesses			
Hypertension	1185 (23.08%)	333 (19.39%)	<0.05
Diabetes	182 (3.54%)	25 (1.46%)	<0.05
Heart disease	520 (10.13%)	134 (7.80%)	<0.05
Stroke, cerebrovascular disease	268 (5.22%)	90 (5.24%)	>0.05
Dyslipidemia	104 (2.03%)	17 (0.99%)	<0.05
Healthy lifestyle group			
Unfavourable	2792 (54.38%)	1083 (63.08%)	<0.05
Favourable	2342 (45.62%)	634 (36.92%)	
Healthy lifestyle factors			
Healthy diet	2818 (54.89%)	828 (48.22%)	<0.05
Never smoking	3884 (75.65%)	1468 (85.50%)	<0.05
Regular physical exercise	1996 (38.88%)	473 (27.55%)	<0.05
Never drinking	3978 (77.48%)	1461 (85.09%)	<0.05
Active cognitive activity	4137 (80.58%)	1049 (61.09%)	<0.05
Active social contact	342 (6.66%)	62 (3.61%)	<0.05
The number of healthy lifestyle factors	3.34 ±1.17	3.11±1.11	<0.05
Socioeconomic status group			<0.05
Lower	2321 (45.21%)	1153 (67.15%)	
Higher	2813 (54.79%)	564 (32.85%)	
Socioeconomic status factors			

Years of education			<0.05
<1	2343 (45.64%)	1244 (72.45%)	
1~6	2006 (39.07%)	382 (22.25%)	
＞6	785 (15.29%)	91 (5.30%)	
Occupation			<0.05
White collar	591 (11.51%)	80 (4.66%)	
Other types	4543 (88.49%)	1637 (95.34%)	
Economic independence			<0.05
One's own	2075 (40.42%)	316 (18.40%)	
Others	3059 (59.58%)	1401 (81.60%)	
Economic status			<0.05
Very rich	61 (1.19%)	16 (0.93%)	
Rich	675 (13.15%)	213 (12.41%)	
General	3611 (70.34%)	1150 (66.98%)	
Poor	667 (12.99%)	284 (16.54%)	
Very poor	120 (2.34%)	54 (3.15%)	
Timely access to healthcare services			<0.05
Yes	4844 (94.35%)	1588 (92.49%)	
No	290 (5.65%)	129 (7.51%)	
Place of residence			<0.05
Urban	956 (18.62%)	270 (15.73%)	
Town	1100 (21.43%)	346 (20.15%)	
Rural	3078 (59.95%)	1101 (64.12%)	
SEVI	0.57 ±0.20	0.66±0.16	<0.05
Follow-up period (months)	76.15±34.34	56.40±29.15	<0.05

Supplementary table 11. Interaction between healthy lifestyle and socioeconomic status on the onset of cognitive impairment using Rubin's method.

	**HR(95%CI)[a]**	**P value**
Healthy lifestyle group		
Unfavourable	Reference	
Favourable	0.823(0.740,0.914)	<0.05
Socioeconomic status group		
Lower		
Higher	0.562(0.502,0.630)	<0.05
Healthy lifestyle *Socioeconomic status		
Favourable*higher	1.201(1.017,1.419)	<0.05
[b]Test for linear trend		
Healthy lifestyle factors	1.049(0.923,1.192)	>0.05
SEVI	2.792(1.339,5.824)	<0.05
Healthy lifestyle factors*SEVI	0.813(0.670,0.986)	<0.05

[a]Adjusted covariates of a time dependent Cox regression model included MMSE score at baseline, age, gender, region, marital status, BMI, BADL disability, IADL disability, hypertension, diabetes, heart disease, stroke& cerebrovascular disease, and dyslipidemia.

[b]Performed by treating healthy lifestyle factors as a numeric variable.

Supplementary table 12. Hazard ratios of SEVI on the onset of cognitive impairment in a population stratified by healthy lifestyle using Rubin's method.

	HR(95%CI)[a]	P value
**All particulars**		
Healthy lifestyle factors	0.936(0.902,0.971)	<0.05
SEVI	2.528(1.910,3.346)	<0.05
**Unfavourable group**		
SEVI	2.975(1.956,4.524)	<0.05
**Favourable group**		
SEVI	1.299(0.907,2.531)	>0.05

[a]Adjusted covariates of a time dependent Cox regression model included MMSE score at baseline, age, gender, region, marital status, BMI, BADL disability, IADL disability, hypertension, diabetes, heart disease, stroke& cerebrovascular disease, and dyslipidemia.

Supplementary table 13 Hazard ratios of socioeconomic status on the onset of cognitive impairment in a population stratified by healthy lifestyle using Rubin's method: subgroup analyses.

	Age[a]		Gender[b]		Marital status[c]	
	<80 (n=3173)	≥80 (n=3678)	Male (n=3347)	Female (n=3504)	Married (n=3133)	Unmarried(n =3718)
**All particulars**						
Healthy lifestyle group						
Unfavourable	Reference	Reference	Reference	Reference	Reference	Reference
Favourable	0.837(0.712, 0.984)*	0.907(0.824, 0.998)*	0.778(0.676, 0.895)**	0.741(0.662, 0.830)**	0.839(0.725, 0.970)*	0.752(0.676,0.837)**
Socioeconomic status group						
Lower	Reference	Reference	Reference	Reference	Reference	Reference
Higher	0.629(0.531, 0.745)**	0.826(0.746, 0.914)**	0.757(0.661, 0.867)**	0.958(0.865, 1.062)	0.728(0.627, 0.847)**	0.910(0.824,1.005)
**Unfavourable group**						
Socioeconomic status						
Lower	Reference	Reference	Reference	Reference	Reference	Reference
Higher	0.680(0.526, 0.878)*	0.797(0.673, 0.944)*	0.679(0.556, 0.829)**	0.820(0.670, 1.003)	0.751(0.599, 0.941)*	0.847(0.705,1.016)
**Favourable group**						
Socioeconomic status						
Lower	Reference	Reference	Reference	Reference	Reference	Reference
Higher	0.860(0.626, 1.181)	0.929(0.759, 1.136)	0.859(0.632, 1.170)	0.874(0.736, 1.039)	0.849(0.663, 1.088)	0.872(0.709,1.073)

[a]Adjusted covariates of a time dependent Cox regression model included MMSE score at baseline, age, gender, region, marital status, BMI, BADL disability, IADL disability, hypertension, diabetes, heart disease, stroke& cerebrovascular disease, and dyslipidemia.

[b]Adjusted covariates of a time dependent Cox regression model included MMSE score at baseline, age, region, marital status, BMI, BADL disability, IADL disability, hypertension, diabetes, heart disease, stroke& cerebrovascular disease, and dyslipidemia.

[c]Adjusted covariates of a time dependent Cox regression model included MMSE score at baseline, age, gender, region, BMI, BADL disability, IADL disability, hypertension, diabetes, heart disease, stroke& cerebrovascular disease, and dyslipidemia.

Supplementary table 14. Association of healthy lifestyle and socioeconomic status with cognitive impairment by adjusting with the death as the competing risk and using the inverse probability weighting method.

	**Model 1**[a]		**Model 2**[b]	
	**HR(95%CI)**[c]	**P value**	**HR(95%CI)**[c]	**P value**
**All particulars**				
Healthy lifestyle group				
Unfavourable	Reference		Reference	
Favourable	0.886(0.799,0.983)	0.023	0.887(0.811,0.970)	<0.05
Socioeconomic status group				
Lower	Reference			
Higher	0.779(0.696,0.872)	<0.05	0.800(0.725,0.883)	<0.05
[b]Test for linear trend				
Healthy lifestyle factors	0.951(0.909,0.996)	0.032	0.941(0.904,0.980)	<0.05
SEVI	2.286(1.591,3.285)	<0.05	1.816(1.335,2.470)	<0.05
**Unfavourable group**				
Socioeconomic status				
Lower	Reference		Reference	
Higher	0.752(0.641,0.882)	<0.05	0.777(0.655,0.920)	<0.05
SEVI	2.736(1.571,4.770)	<0.05	2.165(1.323,3.541)	<0.05
**Favourable group**				
Socioeconomic status				
Lower	Reference		Reference	
Higher	1.048(0.873,1.257)	0.620	0.866(0.725,1.033)	>0.05
SEVI	0.877(0.480,1.601)	0.670	1.02(0.508,2.046)	>0.05

[a]Model 1 adjusted with the death as the competing risk.

[b]Model 2 using the inverse probability weighting method.

[c]Adjusted covariates included MMSE score at baseline, age, gender, region, marital status, BMI, BADL disability, IADL disability, hypertension, diabetes, heart disease, stroke& cerebrovascular disease, and dyslipidemia.

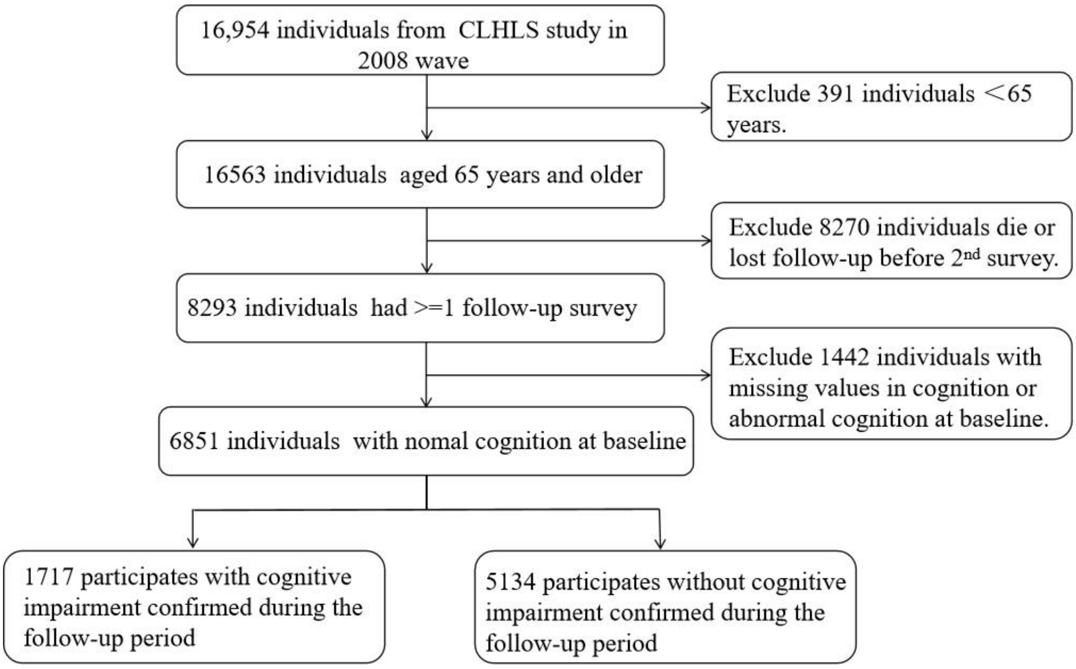

Supplementary figure 1. Flow chart of the included CLHLS individuals.

Characteristics	P value	HR(95%CI)
Healthy diet (Yes)	0.425	1.035(0.952,1.125)
Never smoking (No)	0.269	1.069(0.950,1.203)
Regular physical exercise (Yes)	<0.001	0.772(0.707,0.843)
Never drinking (No)	0.026	1.139(1.016,1.278)
Active cognitive activity (Yes)	<0.001	0.787(0.721,0.859)
Active social contact (Yes)	0.017	0.779(0.635,0.956)
Years of education		
1~6	0.057	1.211(0.994,1.475)
<1	<0.001	1.704(1.396,2.081)
Occupation (other types)	0.443	1.087(0.879,1.343)
Economic independence (Others)	0.001	1.221(1.088,1.370)
Economic status		
Rich	0.317	1.222(0.825,1.808)
General	0.194	1.287(0.879,1.886)
Poor	0.062	1.456(0.982,2.159)
Very poor	0.410	1.207(0.771,1.889)
Timely access to healthcare services (No)	0.814	0.980(0.831,1.156)
Place of residence		
Town	<0.001	0.739(0.647,0.844)
Rural	0.023	0.865(0.764,0.980)

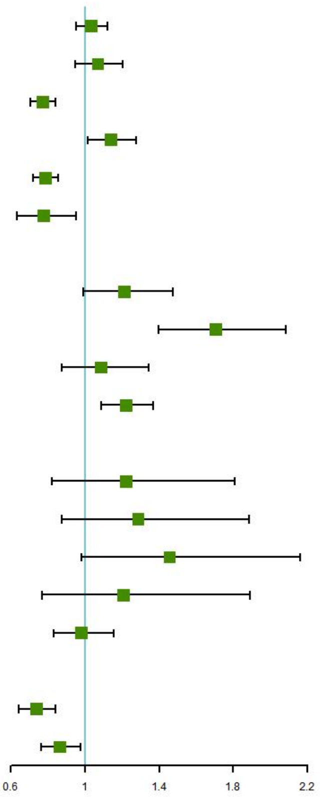

Supplementary figure 2. Hazard ratios of each lifestyle and socioeconomic status factor on the onset of cognitive impairment

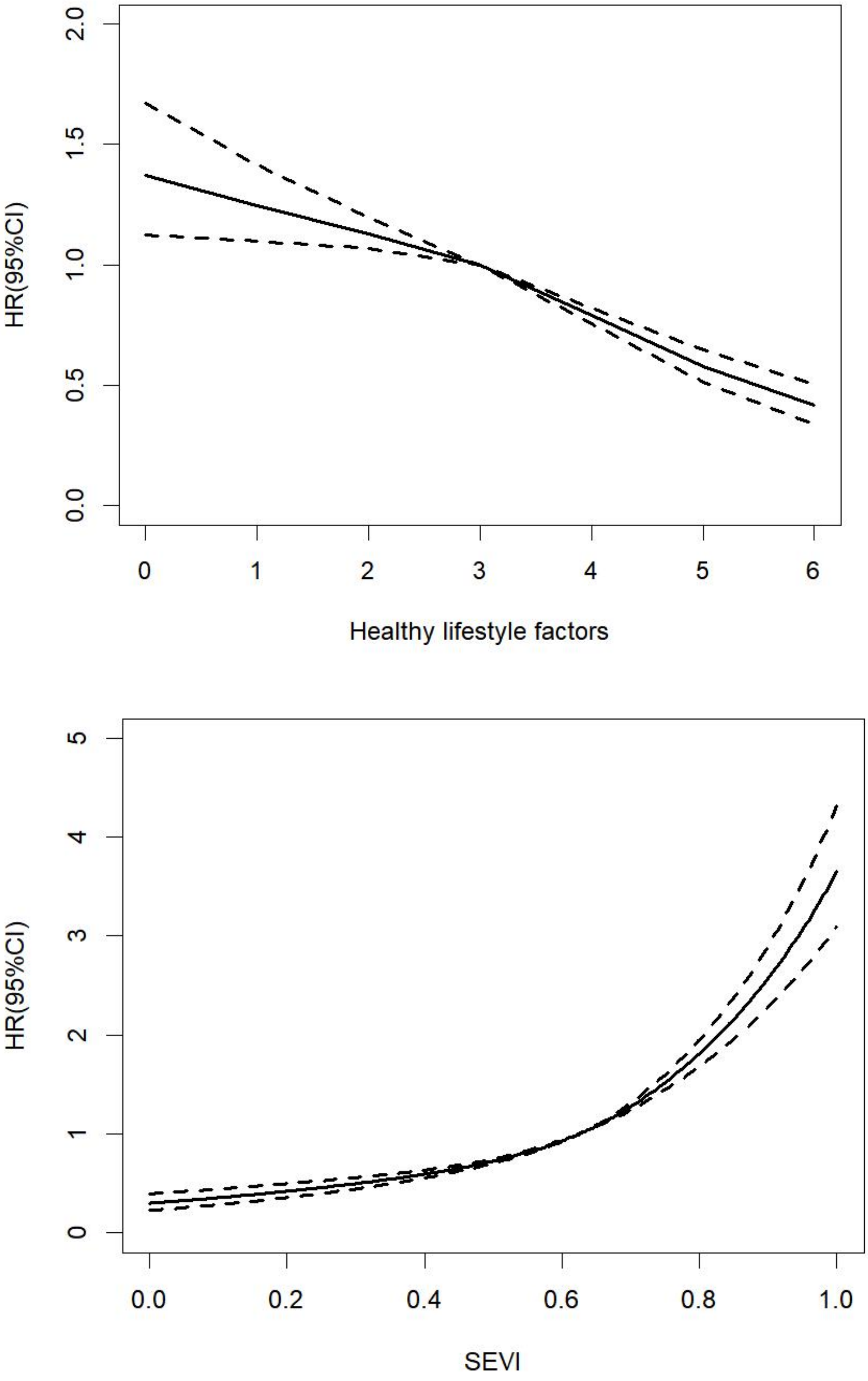

Supplementary figure 3. Cubic splines of the number of healthy lifestyle factors and SEVI on the risk of cognitive impairment.
